# Deleteagene: A Fast Neutron Deletion Mutagenesis-Based Gene Knockout System for Plants

**DOI:** 10.1002/cfg.148

**Published:** 2002-04

**Authors:** Xin Li, Michael Lassner, Yuelin Zhang

**Affiliations:** 1 Biotechnology Laboratory Room 237 6174 University Blvd. University of British Columbia BC V6T 1Z3 Canada; 2 Maxygen, Inc. 515 Galveston Drive Redwood City CA 94063 USA

## Abstract

Deleteagene^™^ (Delete-a-gene) is a deletion-based gene knockout system for plants. To obtain deletion mutants for a specific gene, random deletion libraries created by fast neutron mutagenesis are screened by polymerase chain reaction (PCR) using primers flanking the target gene. By adjusting the PCR extension time to preferentially amplify the deletion alleles, deletion mutants can be identified in pools of DNA samples with each sample representing more than a thousand mutant lines. In *Arabidopsis*, knockout plants for greater than 80% of targeted genes have been obtained from a population of 51 840 lines. A large number of deletion mutants have been identified and multiple deletion alleles are often recovered for targeted loci. In *Arabidopsis*, the method is very useful for targeting small genes and can be used to find deletion mutants mutating two or three tandem homologous genes. In addition, the method is demonstrated to be effective in rice as a deletion mutant for a rice gene was obtained with a similar approach. Because fast neutron mutagenesis is applicable to all plant genetic systems, Deleteagene^™^ has the potential to enable reverse genetics for a wide range of plant species.


**Comparative and Functional Genomics**
*Comp Funct Genom* 2002; **3**: 158–160.
Published online 7 March 2002 in Wiley InterScience (www.interscience.wiley.com). **DOI:** 10.1002/cfg.148

**Conference Review**

### Deleteagene: a fast neutron deletion mutagenesis-based gene knockout system for plants

Xin Li[1], Michael Lassner[2] and Yuelin Zhang[1]*
[1] *Biotechnology Laboratory, Room 237, 6174 University Blvd., University of British Columbia, BC V6T 1Z3, Canada*
[2] *Maxygen, Inc., 515 Galveston Drive, Redwood City, CA 94063, USA*

*Correspondence to: Biotechnology Laboratory, Room 237, 6174 University Blvd., University of British Columbia, BC V6T 1Z3, Canada. E-mail: yuelin@interchange.ubc.ca*

### Abstract

**Deleteagene™ (Delete-a-gene) is a deletion-based gene knockout system for plants. To obtain deletion mutants for a specific gene, random deletion libraries created by fast neutron mutagenesis are screened by polymerase chain reaction (PCR) using primers flanking the target gene. By adjusting the PCR extension time to preferentially amplify the deletion alleles, deletion mutants can be identified in pools of DNA samples with each sample representing more than a thousand mutant lines. In *Arabidopsis*, knockout plants for greater than 80% of targeted genes have been obtained from a population of 51 840 lines. A large number of deletion mutants have been identified and multiple deletion alleles are often recovered for targeted loci. In *Arabidopsis*, the method is very useful for targeting small genes and can be used to find deletion mutants mutating two or three tandem homologous genes. In addition, the method is demonstrated to be effective in rice as a deletion mutant for a rice gene was obtained with a similar approach. Because fast neutron mutagenesis is applicable to all plant genetic systems, Deleteagene™ has the potential to enable reverse genetics for a wide range of plant species. Copyright © 2002 John Wiley & Sons, Ltd.**

Received: 29 January 2002
Accepted: 30 January 2002

**Keywords:** reverse genetics; gene knockout; fast neutron mutagenesis; PCR; deletion mutants; plant functional genomics

### Introduction

The complete genome sequence of *Arabidopsis* has been known for more than a year [1]. Both the public (Chinese Rice Genome Sequence Consortium) and the private (TMRI, Syngenta Inc.) sector reported on draft sequences of the rice genome during the Plant, Animal and Microbe Genomes (PAMG) X Meeting. With the complete genome sequences available for these two model plant species, the challenge for the post-sequencing era becomes the functional characterization of all the genes identified by the sequencing efforts. Reverse genetics will play an essential role in both *Arabidopsis* and rice functional genomics. One general approach in reverse genetics is to create knockouts for target genes and compare the phenotypes of the mutants to wild type plants. In higher plants, since there is no reliable homologous recombination system, creating knockout mutants is not a trivial task.

In *Arabidopsis*, random large-scale insertion mutagenesis has been widely used to inactivate target genes [8]. An insertion in a targeted gene can be identified by a polymerase chain reaction (PCR) using a combination of a gene-specific primer and a primer complementary to the T-DNA, or transposon, border sequences [6]. Alternatively, an insertion in a target gene can be identified by searching a DNA sequence database with a collection of a large number of insertion site sequences, once such a database is established by sequencing the insertion sites of a large number of random insertion lines [7,9]. Although insertion mutants for most *Arabidopsis* genes can be identified using the current collections of insertion lines, knockout plants for a

Copyright © 2002 John Wiley & Sons, Ltd.

significant percentage of genes probably cannot be obtained by this approach. To have a 99% probability of finding an insertion in a 1 kb gene, about 550 000 insertion lines would need to be screened [3].

In *Arabidopsis*, TILLING has been used to screen for EMS-induced point mutations [2]. A small percentage of the identified point mutations completely inactivate the target genes. The main drawback of TILLING is that less than ten plants can be screened in each reaction and the reactions need to be analyzed on a sequencing gel. This greatly limits the throughput and utility of the method. More recently, we reported a high-throughput knockout system based on fast neutron deletion mutagenesis [4]. This method is very effective in obtaining knockout mutants in *Arabidopsis*. We also demonstrated that the same approach could be used in rice.

### General strategies for deleteagene

To obtain deletion mutants for targeted genes, random deletion libraries are produced by fast neutron mutagenesis and then screened for specific deletion mutants by PCR. To construct a deletion library, wild type seeds are treated with fast neutrons and subsequently planted. M2 Seeds from the individual plants are harvested and stored. A small portion of the M2 seeds from each M1 line is planted again. Tissue samples are collected from the M2 seedlings and used to isolate genomic DNA representing the corresponding mutant lines. For PCR screening, DNA samples representing all the mutant lines are aliquoted and organized into pools of increasing complexity. Deletion mutants are first identified in large pools consisting of over a thousand lines. Subsequent deconvolution using the smaller pools leads to identification of the single mutant lines.

To screen an insertion library for a mutant by PCR, a gene specific primer and a primer specific to the insertion element are used. Because only one gene specific primer is used in the PCR, wild type DNA is not amplified. On the other hand, screening for a deletion mutant by PCR uses two primers specific to the targeted locus. Both the wild type gene and the mutant gene will be amplified under normal PCR conditions. In the Deleteagene system, PCR extension time is shortened to suppress the amplification of the wild type fragment. As a result, deletion mutants can be routinely detected in pools of over a thousand lines.

### Applications of Deleteagene in *Arabidopsis*

To characterize the *Arabidopsis* mutant population, 25 loci were screened for deletion mutations in a population containing a total of 51 840 lines. Deletion mutants were identified for 21 of the 25 loci and multiple alleles were identified for most of the target loci. Based on this data, we estimate that a population of 100 000 lines will enable a >95% success rate in isolating deletions in target genes. This high success rate makes Deleteagene an ideal method for knocking out genes that insertion mutations cannot be obtained for. Moreover, isolation of multiple independent alleles for a target gene greatly simplifies the downstream phenotypic characterization process.

One challenge in *Arabidopsis* functional genomics is to find insertions in genes smaller than 1 kb because the probability of finding an insertion in a gene is directly proportional to the size of the gene [3]. Since it is much easier to hit a small gene with a big deletion than with an insertion, Deleteagene will be very useful for isolating knockouts for small genes. For example, we isolated a mutant with a 2.7 kb deletion that completely removed a 0.3 kb target gene. In another example, we obtained a mutant with an 8 kb deletion that completely removed a 0.7 kb target gene.

Another challenge is to determine the functions of genes in tandem arrays. Sequence analysis of the *Arabidopsis* genome revealed 1528 tandem arrays containing 4140 individual genes [1]. If genes in a tandem duplication encode redundant functions, reverse genetic analysis will be difficult because both genes need to be inactivated at the same time in order to observe the mutant phenotype. Using Deleteagene, we can isolate deletions mutating two or three tandem homologous genes. In one example, a mutant with a 9.7 kb deletion that completely removes two closely related bZIP transcription factors was obtained. These two genes are directly linked on Chromosome 5. In another example, a pair of closely linked genes, AOX1a and AOX1b, was found to be completely removed by a 15.7 kb deletion. We also found a single deletion inactivating the three ribulose-1,5-biphosphate carboxylase small subunit genes located on chromosome 5.

Copyright © 2002 John Wiley & Sons, Ltd.

### Application of Deleteagene in crop plants

Since fast neutron mutagenesis can be performed on a large number of dry seeds and no plant transformation is required, Deleteagene can be applied to almost any plant species. In crop plants such as rice, which has large amount of genomic sequence information, Deleteagene can be used very effectively to knockout target genes. On the other hand, it is very difficult to saturate the rice genome with T-DNA or transposable elements since rice has a genome size three to four times larger than *Arabidopsis* and transformation in rice is not nearly as efficient as in *Arabidopsis*. We demonstrated that deletion mutants can be obtained by screening a fast neutron-mutagenized rice mutant population. Expanding the rice fast neutron population should enable us to cover the whole rice genome with easily detectable deletion mutations.

In crop plants, deleting unwanted genes from the existing germlines can also be used as a strategy to generate desirable phenotypes for agriculture. For example, soybeans with reduced concentrations of antinutritional oligosaccarides, stachyose, raffinose, and galactose can be obtained by inactivating genes involved in the synthesis of these compounds [5]. Since no foreign DNA is introduced in the improved crop plants, the resultant crop varieties will not be GMOs and will not face the regulatory or public acceptance barriers associated with transgenic crops.

### References

1. The Arabidopsis Genome Initiative. 2000. Analysis of the genome sequence of the flowering plant *Arabidopsis thaliana*. *Nature* **408**: 796–815.
2. Colbert T, Till BJ, Tompa R, *et al*. 2001. High-throughput screening for induced point mutations. *Plant Physiol* **126**: 480–484.
3. Krysan PJ, Young JC, Sussman MR. 1999. T-DNA as an insertional mutagen in *Arabidopsis*. *Plant Cell* **11**: 2283–2290.
4. Li X, Song Y, Century K, *et al*. 2001. A fast neutron deletion mutagenesis-based reverse genetics system for plants. *Plant J* **27**: 235–242.
5. Mazur B, Krebbers E, Tingey S. 1999. Gene discovery and product development for grain quality traits. *Science* **285**: 372–375.
6. McKinney EC, Ali N, Traut A, *et al*. 1995. Sequence-based identification of T-DNA insertion mutations in *Arabidopsis*: Actin mutants *act2–1* and *act4–1*. *Plant J* **8**: 613–622.
7. Parinov S, Sevugan M, Ye D, *et al*. 1999. Analysis of flanking sequences from *dissociation* insertion lines: a database for reverse genetics in *Arabidopsis*. *Plant Cell* **11**: 2263–2270.
8. Parinov S, Sundaresan V. 2000. Functional genomics in *Arabidopsis*: large-scale insertional mutagenesis complements the genome sequencing project. *Curr Opin Biotechnol* **11**: 157–161.
9. Tissier AF, Marillonnet S, Klimyuk V, *et al*. 1999. Multiple independent defective *Suppressor-mutator* transposon insertions in *Arabidopsis*: A tool for functional genomics. *Plant Cell* **11**: 1841–1852.

Copyright © 2002 John Wiley & Sons, Ltd.
